# Magnetic resonance imaging-guided lumbar nerve root infiltrations: optimization of an in-house protocol

**DOI:** 10.1186/s12880-021-00641-0

**Published:** 2021-07-12

**Authors:** Max Scheffler, Pauline Coralie Guillemin, Orane Lorton, Enrique Maturana, Nicolas Lauper, Dennis E. Dominguez, Sylvain Terraz, Pierre-Alexandre Poletti, Rares Salomir, Sana Boudabbous

**Affiliations:** 1 Division of Radiology, Geneva University Hospitals, Ch. du Pont-Bochet 3, 1226 Thonex, Switzerland; 2 Division of Radiology, University of Geneva, Rue Gabrielle-Perret-Gentil 4, 1205 Geneva, Switzerland; 3 Division of Orthopedic Surgery and Traumatology, Geneva University Hospitals, Rue Gabrielle-Perret-Gentil 4, 1205 Geneva, Switzerland; 4 Division of Radiology, Geneva University Hospitals, Rue Gabrielle-Perret-Gentil 4, 1205 Geneva, Switzerland

**Keywords:** MRI guidance, Nerve root infiltration, Radicular pain

## Abstract

**Background:**

For the treatment of radicular pain, nerve root infiltrations can be performed under MRI guidance in select, typically younger, patients where repeated CT exams are not desirable due to associated radiation risk, or potential allergic reactions to iodinated contrast medium.

**Methods:**

Fifteen 3 T MRI-guided nerve root infiltrations were performed in 12 patients with a dedicated surface coil combined with the standard spine coil, using a breathhold PD sequence. The needle artifact on the MR images and the distance between the needle tip and the infiltrated nerve root were measured.

**Results:**

The distance between the needle tip and the nerve root was 2.1 ± 1.4 mm. The visual artifact width, perpendicular to the needle long axis, was 2.1 ± 0.7 mm. No adverse events were reported.

**Conclusion:**

This technical note describes the optimization of the procedure in a 3 T magnetic field, including reported procedure time and an assessment of targeting precision.

## Background

The burden of low back pain is substantial with an overall global prevalence estimated at 9%, making it one of the leading causes of disability [1, 2]. The etiology of low back pain is mostly degenerative and multifactorial. Degenerative changes not only trigger localized pain but may also give rise to radicular pain in the buttocks and lower limbs. It has been estimated that approximately 90% of sciatica, a common form of radicular pain, is due to disc herniation [3].

A number of interventional and surgical options are available for the treatment of radicular pain that is intractable to conservative medical management (including physical therapy), or is characterized by recurrence, with percutaneous infiltrations being the least invasive option. Infiltrations are typically performed under fluoroscopy, computed tomography (CT) or real-time CT guidance [4]. For CT-guided infiltrations a reported mean effective dose is 1.38 mSv [5].

The age of onset of sciatica may occur earlier than low back pain, with a peak incidence thought to occur in patients in their forties [6]. Repeated CT- or fluoroscopy-guided infiltrations, requested within an interval of a few months in younger patients, may be problematic from a radioprotection perspective. Furthermore, if the operator opts for an injection of iodinated contrast medium around the nerve, the patient is exposed to a risk of a contrast-induced allergic reaction, even if a very small amount is used. Magnetic resonance imaging (MRI) guidance in 1 T and 1.5 T magnetic fields has been used as a feasible and safe alternative [7–9]. MRI is free of ionizing radiation and offers a high soft tissue and fluid contrast but comes with the disadvantages of higher costs and a longer imaging time compared to CT [10].

We describe herein our MRI-guided infiltration method on a 3 T machine for the treatment of radicular pain. We quantitatively assessed the inclination of the MRI-conditional needle, the distance between the needle tip and the targeted nerve root, and the artifact created by the needle.

## Methods

Fifteen MRI-guided nerve root infiltrations were carried out in 12 patients (mean age, 57 years ± 18 SD; 6 females) on a 3 T MRI machine (Magnetom Skyra, Siemens Healthineers, Erlangen, Germany) between November 2017 and January 2021. Patients were consecutively selected by an expert board.

**Fig. 1:**
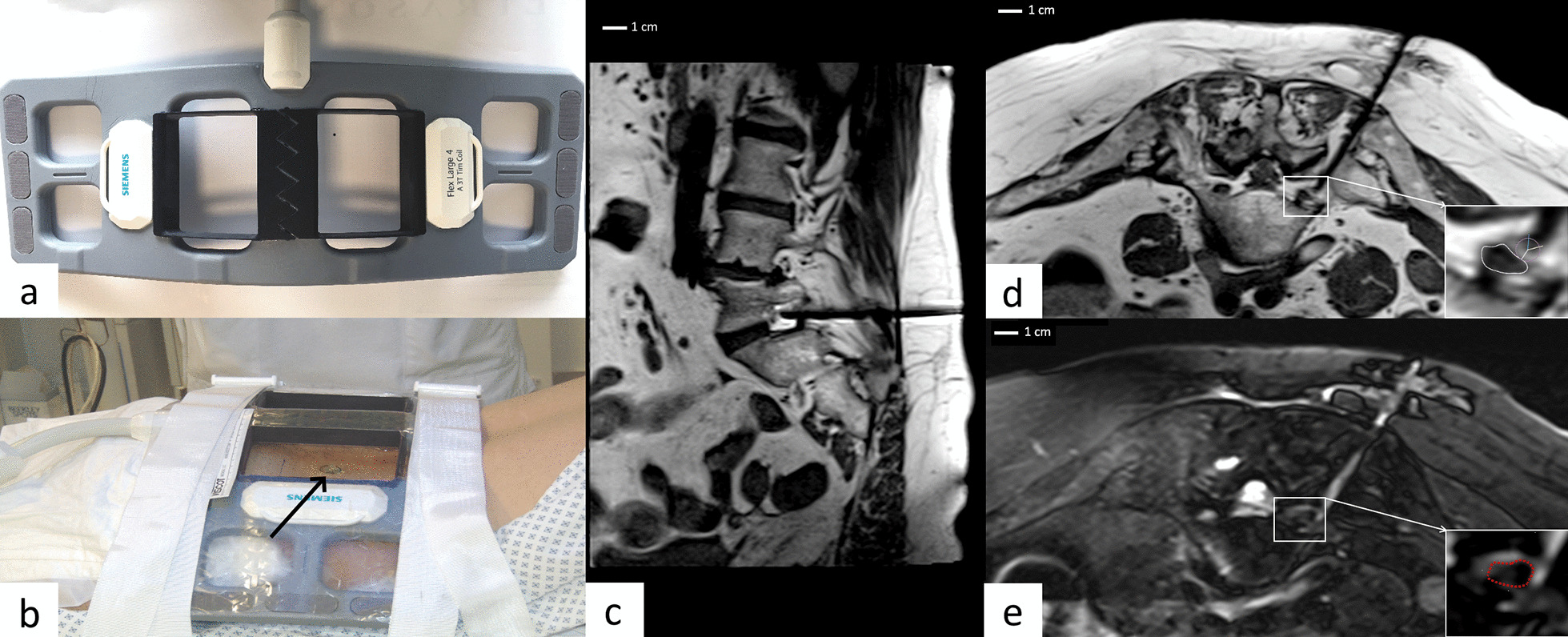
**a** Photograph of the adapted commercial coil. The four medial openings have been merged into two larger ones, the cut edges sealed and reinforced by rigid polymer frames produced on a 3D printer. **b** Setup of the patient positioned in the prone position on the MRI table, with the flexible coil placed on the lower back. A small silicone marker (arrow) is placed on the skin entry point inside the coil opening before acquisition of confirmatory images. The coil is attached by fastening straps. **c**, **d** Intraprocedural proton density (PD) original (not reconstructed) sagittal oblique and axial images, showing the needle in its final position behind an exiting L5 nerve root, within the neural foramen, more clearly seen on the image inset encircled by white (**d**) freehand region of interest (ROI). The needle tip location is confirmed behind an exiting L5 nerve root, within the neural foramen; **e** Heavily T2-weighted fat-saturated (FS) axial image acquired after the injection of a small quantity of sterile saline solution, seen as a signal hyperintensity surrounding the nerve root, encircled by red freehand ROI in image inset

After confirmation of written consent, patients were placed on the MR table in a prone position. A dedicated surface coil proved to be necessary in addition to the standard spine coil. For this reason, we adapted a commercially available flexible surface coil (4-Channel Flex Large Coil, Siemens Healthineers, Erlangen, Germany) (Fig. 1a) in order to provide an enlarged entry window. The four central openings among the total of eight openings in the coil foam matrix were merged into two larger ones. The cut edges were resealed with epoxy resin and the openings reinforced by rigid polymer frames produced on a 3D printer, without modification of the radiofrequency resonant circuit. The widened openings offered sufficient manipulation space, even when the needle was significantly tilted. The coil was placed in the lumbar region, perpendicular to the long body axis, and one of the two large windows centered over the presumed point of skin puncture. The coil was attached with fastening straps to the MR table (Fig. 1b).

Standard T2-weighted turbo spin echo (TSE) sagittal and axial sequences were acquired for access planning. Main parameters of this sequences were: TE, 103 ms; TR, 2300 ms; flip angle, 150°; bandwidth, 260 Hz/pixel; acquisition matrix, 256 × 140; FOV, 300 × 300 mm; number of averages, 1; slice thickness, 2.5 mm; acquisition time (AT), 14 s (one breathhold). Sagittal images confirmed the correct level to be infiltrated. A small silicone marker (Ø = 6 mm, MR-Pinpoint 187, Beekley Medical, Bristol, USA) was placed on the chosen skin entry point and the coil position adjusted if necessary. In all cases we opted for a projected needle path where the nerve root could be reached as medially as possible, ideally inside in the neural foramen, in a strict axial plane.

The skin area under the coil’s window was disinfected and covered with a perforated sterile film dressing. A layer of sterile tissue drapes was used to cover the entire lower back area of the patient. Superficial and deep local anesthesia was performed with 1% lidocaine solution (Rapidocain 1%, Sintetica, Mendrisio, Switzerland). Controlled by repeated proton density (PD)-weighted multi-slice axial and sagittal oblique breathhold acquisitions, an MRI-conditional 20G needle with a stylet (external diameter 0.9 mm, Cytocut MRI, MDL, Delebio, Italy) was inserted and directed towards the exiting nerve root (Fig. 1c, d), aiming for an as short as possible needle-tip-nerve root distance. In some cases, a radiologic technologist facilitated adjustment of the needle’s angulation with the assistance of a goniometer. The final position was determined intra-procedurally by the operator, taking into account possible radicular pain reported by the patient, always warranting a slight retreat of the needle. The PD sequence used for needle guidance had the following parameters: TE, 9.1 ms; TR, 1030 ms; flip angle, 132°; bandwidth, 199 Hz/pixel; acquisition matrix, 256 × 252; FOV, 240 × 240 mm; number of averages, 2; slice thickness, 2 mm; acquisition plane, axial or sagittal oblique; acquisition time (AT), 17 s (one breathhold). To reduce imaging time, the slice stack coverage was limited to 11.6 mm (five 2 mm-thick slices with a distance factor of 0.4 mm). The selected phase encoding direction was anterior–posterior in order to minimize the chemical shift-like artifacts of the needle tip projecting along the readout direction.

With the needle tip positioned as close as possible behind the exiting nerve root and after a first attempt of aspiration, 1–2 ml of sterile saline solution (NaCl 0.9%) were injected. A heavily T2-weighted fat-saturated single breathhold SPAIR (SPectral Attenuated Inversion Recovery) sequence was immediately performed to confirm the extravascular position of the needle tip and to visualize periradicular, and often epidural, diffusion of the fluid (Fig. 1e). This sequence had the following parameters: TE, 104 ms; TR, 2420 ms; flip angle, 150°; bandwidth, 260 Hz/pixel; acquisition matrix, 192 × 154; FOV, 128 × 128 mm; number of averages, 1; slice thickness, 3 mm; AT, 18 s (one breathhold). If no fluid was visible on the SPAIR images, an intravascular position of the needle was thought to be likely and the needle moved back slightly, to inject another small amount of sterile saline.

After repeating the aspiration, injection of the active medication was performed. According to the preference of the requesting physician, we used 1 ml of dexamethasone 0.4% solution (Mephameson 4 mg/ml, Mepha Pharma, Basel, Switzerland) ± 1 ml of ropivacaine 0.5% solution (Ropivacain 5 mg/ml, Sintetica, Mendrisio, Switzerland).

Two metrics were used to assess the needle artifact on MR images. The first one consisted of a computer-based standardized measurement which did not correspond to the actual image guidance impairment but enabled a through-patient quantitative comparison. For this purpose, a 1D signal profile from the proton density axial image, cutting the needle perpendicularly, was approximated as an inversed Gaussian curve and the full width at half maximum (FWHM) was determined. The second metric used to evaluate the visual artifact, considered the subjective user-defined width of the dark band that artificially increased the needle geometric width.

The distance between the needle tip and the nerve root was calculated retrospectively on the most recent PD image prior to injection, aiming to demonstrate accurate targeting. The farthest left needle pixel, the farthest right needle pixel, and the lowermost needle pixel were used as starting points to draw individual lines to the posterior border of the nerve, with confirmation provided by the operators (M.S., S.B.) in doubtful cases. Procedure times were analyzed through measured room occupation times that included marking of the skin entry point and preparation of the sterile field.

## Results

Results of the targeting end-point MRI data are shown in Table 1. The mean needle artifact according to the first metric was 3.8 mm (SD, 0.9 mm). The mean needle artifact according to the second metric was 2.1 mm (SD, 0.7 mm). The mean measured distance between the needle tip and the nerve root was 2.1 mm (SD, 1.4 mm). The values were compared to the geometric diameter of the needle, 0.9 mm, the ratios are provided in Table 1. Periradicular ± epidural diffusion of the injected normal saline could be observed in all patients before injection of the anti-inflammatory ± anesthetic agents. Mean needle inclination was 19° (SD, 8°). Mean MRI room occupation time was 51 min. The quality of the images enabled increased confidence in technical success in all cases, independent of body habitus and despite the presence of severe spondylolisthesis in one patient. No patient experienced an adverse event during, or after, the procedure.

**Table 1:** Quantitative results of 15 procedures in twelve patients, chronological order

No	Needle-nerve root distance (mm)	Needle angulation (°)	MRI room occupation time (min)	Nominal needle diameter (mm)	FWHM needle artifact (mm)	FWHM needle artifact ratio*	Visual artifact (mm)	Visual artifact ratio
1	2.62	25.0	70	0.9	5.16	5.73	3.66	4.07
2	3.56	27.9	55	0.9	4.94	5.49	3.06	3.40
3	0.99	22.5	60	0.9	4.49	4.99	1.35	1.50
4	1.95	22.7	65	0.9	3.98	4.42	2.68	2.98
5	3.89	16.6	55	0.9	4.73	5.26	1.49	1.66
6	2.69	13.0	54	0.9	4.73	5.26	2.34	2.60
7	1.41	8.2	50	0.9	3.14	3.49	2.99	3.32
8	4.73	21.2	64	0.9	2.57	2.86	1.54	1.71
9	3.20	6.9	49	0.9	3.55	3.94	1.71	1.90
10	0.10	24.6	40	0.9	4.84	5.38	1.83	2.03
11	1.39	33.4	40	0.9	3.75	4.17	2.11	2.34
12	1.89	14.3	39	0.9	3.04	3.38	2.12	2.36
13	1.87	27.2	41	0.9	2.81	3.12	2.02	2.24
14	0.10	12.4	44	0.9	3.05	3.39	1.49	1.66
15	0.83	11.2	40	0.9	2.81	3.12	1.42	1.58
Mean	2.08	19.1	51		3.8	4.27	2.12	2.36
SD	1.36	7.9	11.9		0.91	1.01	0.69	0.78

*SD* standard deviation, *FWHM* full width at half maximum

*Relative to physical diameter of the needle

## Discussion

In our series of fifteen 3 T MRI-guided interventions, good image quality and technical procedural success could be achieved in all cases, irrespective of individual body habitus. The adapted surface coil allowed for standard-of-care disinfection, facilitated access, and was resistant to cleaning with conventional disinfecting surface agents.

Despite some unavoidable susceptibility artifact from the needle, the distance between the tip and the exiting nerve root could be correctly determined. The transversal artifact of approximately 2 mm average width reported here for a 3 T MRI setup was significantly smaller than previously described in the literature at lower magnetic fields, for instance 6 ± 0.2 mm for a 21G needle on a 1.5 T machine [7] and a range of 1.5–5 mm for a 20G needle on a 1 T machine [8]. The negligible longitudinal needle tip artifact was attributed to the fact that the phase encoding direction in the PD axial images avoided the chemical shift-like artifact falsely prolonging the needle. Cases of our series showed distances between the needle tip and the nerve root of up to 4.7 mm. The larger values could be explained by either clinical factors requiring a larger security margin independently of the intrinsic targeting accuracy (e.g. radicular pain signaled by the patient excluding further needle advancement), or an initially intravascular position of the needle tip that warranted a slight retreat. Some operators may prefer a 22G needle when performing a nerve root infiltration—while being less traumatic it may also be more prone to flexion and deviation from a chosen access path—and smaller MR artifacts should be expected in that case, although this also depends on the manufacturing alloy.

The room occupancy time decreased over the period of the 15 procedures, with 40 min needed for the last intervention, in comparison to 70 min for the first. As 3 T MRI enables the performance of very rapid PD sequences (within one single breathhold), the main axis of improvement lies in the patient's set-up time and the orchestration of single steps of the infiltration procedure itself. With select patients we can expect to reach an MRI room occupancy time between 25 and 35 min.

Contrarily to CT, MRI scanners do not have an inclinable gantry but offer freely definable acquisition planes. In our series however, all procedures could be realized in a strict axial plane, with a mean needle angulation of 19.1°.

## Conclusion

In conclusion, for a select group of patients suffering from radicular pain, lumbar nerve root infiltrations could be performed radiation-free and without iodinated contrast medium using 3 T MRI. Our method offers an optimized procedural workflow with high image quality which can be performed within reasonable times and without compromising safety.

## Data Availability

The images of Fig. 1 can be obtained as DICOM files from the corresponding author.

## Abbreviations

CT: Computed tomography
MRI: Magnetic resonance imaging
PD: Proton density
SD: Standard derivation
FWHM: Full width at half maximum
FOV: Field of view
AT: Acquisition time
TR: Repetition time
TE: Echo time
TSE: Turbo spin echo
SPAIR: SPectral Attenuated Inversion Recovery
ROI: Region of interest
